# The Contribution of Dental Amalgam to Urinary Mercury Excretion in Children

**DOI:** 10.1289/ehp.10249

**Published:** 2007-06-28

**Authors:** James S. Woods, Michael D. Martin, Brian G. Leroux, Timothy A. DeRouen, Jorge G. Leitão, Mario F. Bernardo, Henrique S. Luis, P. Lynne Simmonds, John V. Kushleika, Ying Huang

**Affiliations:** 1 Department of Environmental and Occupational Health Sciences, University of Washington, Seattle, Washington, USA; 2 Department of Oral Medicine, University of Washington, Seattle, Washington, USA; 3 Department of Dental Public Health Sciences, University of Washington, Seattle, Washington, USA; 4 Faculdade de Medicina Dentaria, Universidade de Lisboa, Lisbon, Portugal

**Keywords:** amalgam, children, dental, mercury, urine

## Abstract

**Background:**

Urinary mercury concentrations are widely used as a measure of mercury exposure from dental amalgam fillings. No studies have evaluated the relationship of these measures in a longitudinal context in children.

**Objective:**

We evaluated urinary mercury in children 8–18 years of age in relation to number of amalgam surfaces and time since placement over a 7-year course of amalgam treatment.

**Methods:**

Five hundred seven children, 8–10 years of age at baseline, participated in a clinical trial to evaluate the neurobehavioral effects of dental amalgam in children. Subjects were randomized to either dental amalgam or resin composite treatments. Urinary mercury and creatinine concentrations were measured at baseline and annually on all participants.

**Results:**

Treatment groups were comparable in baseline urinary mercury concentration (~ 1.5 μg/L). Mean urinary mercury concentrations in the amalgam group increased to a peak of ~ 3.2 μg/L at year 2 and then declined to baseline levels by year 7 of follow-up. There was a strong, positive association between urinary mercury and both number of amalgam surfaces and time since placement. Girls had significantly higher mean urinary mercury concentrations than boys throughout the course of amalgam treatment. There were no differences by race in urinary mercury concentration associated with amalgam exposure.

**Conclusions:**

Urinary mercury concentrations are highly correlated with both number of amalgam fillings and time since placement in children. Girls excrete significantly higher concentrations of mercury in the urine than boys with comparable treatment, suggesting possible sex-related differences in mercury handling and susceptibility to mercury toxicity.

Much attention has focused on potential adverse health effects associated with exposure to mercury and mercury compounds. Of particular public health concern has been possible neurologic impairment associated with prolonged exposure to elemental mercury (Hg^0^) vapor (Clarkson 2003; Echeverria et al. 1998; Goering et al. 1992). Children are known to be particularly vulnerable to Hg^0^, prolonged exposure to which may cause impairment of the developing central nervous system, along with attendant personality, motor function, and behavioral disorders (Counter and Buchanan 2004; Davidson et al. 2004; Levy et al. 2004).

A principal source of Hg^0^ exposure in children is through dental amalgam fillings, which are approximately 50% metallic mercury by weight. Hg^0^ vapors released from amalgam fillings in tooth surfaces are readily absorbed into the systemic circulation by inhalation (Berglund et al. 1988; Mackert and Berglund 1997; Svare et al. 1981; Vimy and Lorscheider 1985). Once absorbed, Hg^0^ undergoes biotransformation predominantly in erythrocytes, to mercuric ion (Hg^2+^), the ultimate mediator of mercury toxicity (Halbach and Clarkson 1978; Magos et al. 1978). Debate continues as to the potential adverse effects of low-level Hg^0^ exposure from dental amalgam, particularly in children (Brownawell et al. 2005; Clarkson and Magos 2006).

Findings from two concurrently conducted clinical trials that were designed to evaluate the potential neurologic and neurobehavioral consequences of prolonged Hg^0^ exposure from dental amalgam fillings in children have been recently reported (Bellinger et al. 2006; DeRouen et al. 2006). As part of one of those clinical trials (DeRouen et al. 2006), we performed annual measurements of urinary mercury concentrations in children between 8 and 18 years of age as an assessment of longitudinal exposure to Hg^0^ from amalgam fillings. Here we describe changes in urinary mercury levels in children with and without amalgam fillings over the course of the trial. We also report age-, race-, and sex-related changes in urinary mercury concentrations associated with amalgam exposure.

## Methods

### Description of the study population and details of dental treatments

Five hundred seven children (54% boys, 46% girls) 8–10 years of age at baseline who were residents of the Casa Pia school system in Lisbon, Portugal, participated in a randomized, prospective clinical trial to examine the potential health effects of exposure to dental amalgam tooth filling materials. Children were evaluated at baseline and at seven subsequent annual intervals after initial dental treatments with an extensive battery of neurobehavioral, neurologic, and renal function assessments. The dental materials used in this trial (amalgam or composite resin) were state-of-the art, universally accepted tooth filling materials. The choice of amalgam was the brand most widely used in the United States, Dispersalloy by Dentsply Caulk (York, PA, USA), which, like most other brands, is approximately 50% Hg^0^. All dental treatments met existing standards of care in the United States and Portugal. The study protocol was approved by the institutional review boards at the University of Washington and the University of Lisbon. All parents or guardians gave written informed consent, and all children provided signed assent. A detailed description of the demographics of the study population as well as the design and methods employed has been previously published (DeRouen et al. 2002).

### Procedures for urine collection and measuring urinary Hg and creatinine

A urine sample (~ 50 mL) was collected from each child at baseline and at each subsequently scheduled annual visit to the University of Lisbon School of Dental Medicine for dental, neurologic, and neurobehavioral evaluations. Immediately after urine collection, a 10-mL aliquot was removed and acidified with 1 N HCl. Analysis of total mercury was performed using continuous flow, cold vapor spectrofluorometry as previously described (Pingree et al. 2001b). Urinary creatinine concentrations were measured in unacidified urine using a standard colorimetric procedure (Sigma #555-A; Sigma-Aldrich, St. Louis, MO, USA). Urinary mercury levels were calculated as both micrograms per gram creatinine and micrograms per liter urine.

### Statistical procedures

**Figure 1:**
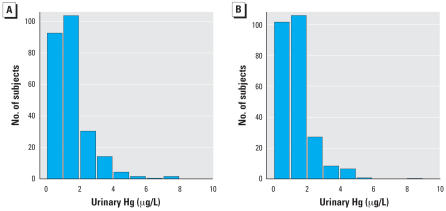
Histograms of baseline urinary mercury concentrations in amalgam (*A*) and composite (*B*) treated groups. Heights of the bars represent the numbers of subjects with values within the indicated range. The distributions of baseline urinary mercury levels were similar in the two treatment groups.

As a check on the randomization procedure, we compared the two treatment groups (amalgam or composite) in terms of the distributions of sex, race, and baseline values of age, urinary mercury concentration, urinary creatinine concentration, blood lead concentration, and IQ (Figure 1 and Table 1). Mean values of mercury concentration and creatinine-adjusted mercury concentration were graphed by treatment group and year, and treatment group means were compared at each follow-up year using *t*-tests. We used arithmetic means for these analyses because of their ease of interpretations; analyses of geometric means (not shown) gave qualitatively similar results. We compared treatment group means first for all study participants and then separately for male and female participants. We compared males and females within the amalgam group using *t*-tests at each study year. The amount of amalgam treatment was characterized by the number of amalgam surfaces placed at baseline (i.e., within 1 year from the first visit) and during the follow-up period. Amalgam group participants were classified according to baseline treatment (0–4, 5–9, or > 9 surfaces) and follow-up treatment (0, 1–9, or > 9 surfaces) (Table 2). Mean urinary mercury concentrations for each of the resulting nine subgroups were then compared with means for the composite group as a whole. We used linear regression analysis to examine the prediction of creatinine-adjusted urinary mercury concentration (on the logarithmic scale) as a function of sex, race, baseline age, study year (as categorical variable), number of amalgam surfaces placed in first year, number of amalgam surfaces placed in subsequent years, and number of amalgam surfaces placed at baseline that were subsequently lost due to tooth exfoliation or extraction. An additional analysis used weighted counts of surfaces placed and lost, weighted for size of restoration (1 = small, 2 = medium, 3 = large), which gave qualitatively very similar results (not reported). The method of generalized estimating equations (Liang and Zeger 1986) was used for this analysis to account for correlation between observations on the same subject in different years. The statistical analyses used all available data, and missing data on children who were not followed were ignored. The main study conclusions were not affected heavily by missing data, because the latter pertain to the initial 5 years of follow-up when missing was infrequent.

Statistical analyses were performed using the statistical package R (version 2.4.1; R Foundation for Statistical Computing, Vienna, Austria).

## Results

### Baseline comparisons of treatment groups

The treatment groups were similar in distribution of sex, race, baseline age, and baseline urinary mercury concentration (Table 1). The distributions of baseline urinary mercury concentrations were very similar in the two treatment groups (Figure 1). The groups were also balanced on other baseline variables examined (DeRouen et al. 2006) including average creatinine-adjusted urinary mercury concentration (1.8 μg/g for the amalgam group, and 1.9 μg/g for the composite group), IQ score (85 and 85), blood lead concentration (4.7 and 4.5 μg/dL), number of carious surfaces (15.6 and 15.9), and creatinine-adjusted albumin concentration (8.6 and 8.3 mg/g).

### Follow-up mercury concentrations

**Figure 2:**
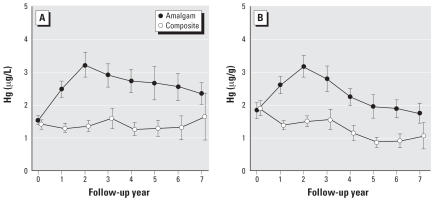
Mean urinary mercury concentrations, unadjusted (*A*) and creatinine-adjusted (*B*), for the amalgam group and composite group. Error bars show 95% confidence intervals for the group means. Group differences were highly statistically significant (*p* < 0.001) for both measures at follow-up years 2 through 6. The group differences at year 7 were not significant for unadjusted mercury (*p* = 0.07) but significant for adjusted mercury (*p* = 0.007).

Mean urinary mercury concentrations in the amalgam group increased from approximately 1.5 μg/L at baseline to a peak of approximately 3.2 μg/L at year 2 and then slowly declined to near baseline levels by year 7 of follow-up (Figure 2). In contrast, mean mercury concentrations changed very little in the composite group throughout the 7-year follow-up period. Differences between treatment groups were highly significant at all follow-up years except for the final year. For creatinine-adjusted mercury levels, group differences were significant at all follow-up years, including year 7 (Figure 2B). A possible reason for the lack of significance at year 7 for unadjusted concentrations is the wide confidence interval due to reduced sample size and a small number of large values in the composite group that increased the group mean and SD.

### Race comparisons

Mean urinary mercury concentrations for black and white participants were similar at baseline as well as throughout all 7 years of follow-up. No significant differences were found by race in urinary mercury concentrations associated with amalgam exposure (not shown).

### Sex comparisons

**Figure 3:**
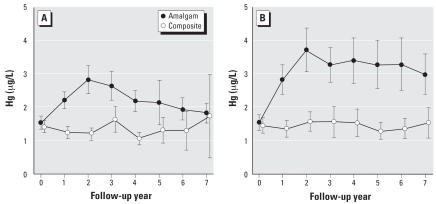
Mean urinary mercury concentrations for the amalgam group and composite group separately for male (*A*) and female (*B*) participants. Error bars show 95% confidence intervals for the group means. Differences between males and females in the amalgam group were statistically significant (*p* < 0.05) at all follow-up years except follow-up year 3. The sex comparisons were not altered significantly by adjustment for creatinine (results not shown).

Mean urinary mercury concentrations for male and female participants were similar at baseline, but increases after amalgam treatment were larger for females than for males. As shown in Figure 3, females who received amalgam fillings had significantly higher mean urinary mercury concentrations than males throughout the 7 years of follow-up. In contrast, there were no differences in urinary mercury concentrations between males and females in the composite group. Mean mercury concentrations for female amalgam group subjects reached a peak of approximately 3.5 μg/L at year 2 and remained about 3 μg/L throughout the 7-year follow-up period. In contrast, mean mercury values for males were < 3 μg/L at all years and declined to the same level as seen in the composite group by the end of follow-up. The differences between males and females in urinary mercury levels were not attributed to the amount of treatment received. As indicated in Table 2, the distributions of amalgam surfaces placed during baseline and follow-up were similar for males and females.

### Dose–effect relationships

**Figure 4:**
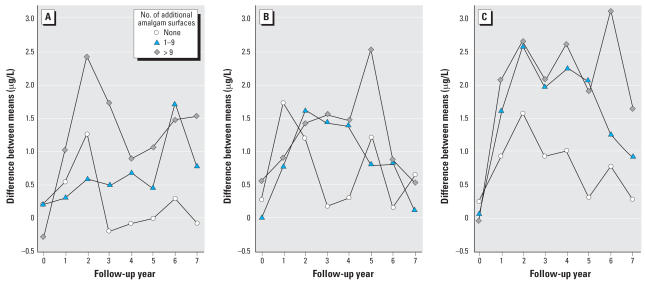
The increase in urinary mercury concentration is influenced by both the amount and timing of amalgam treatment. Children in the amalgam group were categorized according to the number of amalgam surfaces placed at baseline—(*A*) 0–4; (*B*) 5–9; (*C*) > 9—and the number of additional amalgam surfaces placed in subsequent years. The values plotted are the differences between mean urinary mercury in a particular subgroup of amalgam-treated children compared with mean urinary mercury concentration in the composite group at each year.

The increase in urinary mercury concentrations was positively associated with the amount of amalgam treatment received at baseline and during follow-up (Figure 4). The largest increases in mercury concentrations (reaching 3.1 μg/L in year 6) were observed in participants receiving more than 9 amalgam surfaces at baseline and an additional 10 or more surfaces during follow-up. In contrast, only small increases in urinary mercury concentrations were observed in participants receiving 0–4 amalgam surfaces at baseline.

### Regression analysis

In regression analysis, child sex (*p* < 0.001), baseline amalgam surfaces (*p* < 0.001), surfaces lost (*p* < 0.001), and follow-up amalgam surfaces (*p* < 002) were significant predictors of creatinine-adjusted urinary mercury. Concentrations for females were approximately 30% higher than those for males [calculated as exp(0.25), where 0.25 was the difference on the log scale]. Each additional baseline surface was associated with a 0.057 increase in concentration on the log scale (corresponding to about a 6% increase in concentration). Each lost surface was associated with an increase on 0.047 on the log scale; the difference 0.057–0.047 represents the effect of a surface placed and then lost on urinary mercury concentration. Each additional follow-up surface was associated with an increase of 0.018 on the log scale. The effects of age and race were not statistically significant.

## Discussion

Numerous studies have described the relationship between mercury exposure from dental amalgam restorations and its corresponding excretion in the urine of adults (Begerow et al. 1994; Dye et al. 2006; Kingman et al. 1998; Mackert and Berglund 1997; Skare and Engqvist 1994) as well as children (Gearhart et al. 1995; Khordi-Mood et al. 2001; Levy et al. 2004; Pesch et al. 2002; Suzuki et al. 1993). To our knowledge, this is the first study to describe urinary mercury excretion patterns in children during the longitudinal course of amalgam treatment from childhood through adolescence and to quantify the relationship between amalgam surfaces and urinary mercury concentrations during the course of treatment. The findings demonstrate a strong, positive association between urinary mercury concentration and both number of amalgam surfaces and time since placement. Urinary mercury levels were highest approximately 2 years after initial amalgam treatment, regardless of number of surfaces, among children receiving no additional fillings. Among children receiving up to 9 initial amalgam fillings, urinary mercury concentrations returned to pretreatment values within one year, consistent with a whole-body biological half-time of mercury on the order of 60–70 days (Clarkson et al. 1988). In contrast, for children receiving ≥10 amalgam fillings at baseline and with no subsequent treatment, the decline from peak to pretreatment urinary mercury concentrations occurred over a period of ≥3 years, consistent with the kinetics of a two-compartment model of urinary mercury elimination that predicts a substantially longer whole-body mercury half-time (Barregård et al. 1992).

For children receiving > 9 additional amalgam fillings after initial amalgam placement, urinary mercury concentrations remained elevated 2- to 4-fold compared with those of composite controls throughout much of the 7-year follow-up, declining only gradually during this period. This was true regardless of the number of amalgam fillings placed at baseline. Nonetheless, data presented in Figure 2 imply that the rate of urinary mercury excretion exceeds the rate of mercury exposure from dental amalgam in these subjects at all time points. Notably, we observed a constant but quantifiable urinary mercury excretion among children in this study who did not receive amalgam restorations, most likely representing the systemic uptake of mercury from food, air, and other environmental sources. Together, these observations imply that the level of mercury exposure from all sources including amalgam fillings did not exceed the capacity for elimination via urinary excretion in these subjects. That urinary output increases approximately 1.5-fold between 10 and 15 years of age from approximately 1,000 to approximately 1,500 mL/24 hr (Forfar and Arneil 1984) possibly contributes to this capability, although previous reports have suggested that the 24-hr urinary mercury excretion rate is not significantly influenced by the urinary flow rate (Skare and Engqvist 1990, 1994).

Of particular interest was the finding of significantly higher urinary mercury concentrations in girls compared with boys beginning with the first year after initial amalgam placements and continuing through the subsequent 7-year follow-up period. These differences held up after adjustment for creatinine and differences in the amount of amalgam treatment received (Table 2). Factors possibly accounting for this sex difference include differences in *a*) eating habits, particularly total time spent eating, and consumption of hot beverages (Brune 1988), *b*) habitual gum chewing (Gay et al. 1979), *c*) exercise that results in high rates of breathing (Brune 1988), and *d*) body weight (Levy et al. 2004). Variation in eating habits is not likely to contribute to sex differences observed in the present study, in that most subjects were enrolled in the Casa Pia school system, which provided the same meals to all children. Importantly, fish consumption among participants in this study was comparable and did not constitute a significant source of mercury exposure (Evens et al. 2001). Similarly, habitual gum chewing, defined as chewing gum for the greater part of every day, was relatively uncommon within this population and not likely to account for the sex differences observed. In terms of body mass differences, Levy et al. (2004) reported a significant inverse relationship of urinary mercury concentration for children stratified on physical characteristics such as height and weight. However, no sex differences in urinary mercury excretion were reported in that investigation. Although height and weight data were not collected in the present study, no significant differences between boys and girls in creatinine excretion—a surrogate measure of body mass—were found over the course of follow-up (Martin MD, unpublished data), suggesting a more predominant effect of sex per se as opposed to body size or exercise rates on the mercury excretion differences observed in this study.

Sex differences in mercury handling in both animals and humans have been described. In terms of inorganic mercury, Hultman and Nielsen (2001) reported significantly greater whole-body mercury retention as well as greater mercury accumulation in kidneys and spleens of male compared with female mice of several strains during prolonged exposure to mercuric chloride. In human studies, women were reported to have significantly higher urinary mercury concentrations than men with comparable numbers of dental amalgam fillings (Akesson et al. 1991), similar to findings here. Studies on the excretion of organic and inorganic mercury in methylmercury-treated rats (Thomas et al. 1987) showed faster whole-body clearance of mercury in females than in males, also consistent with the present findings. Similarly, studies on methyl-mercury exposure in human infants and children (Grandjean et al. 1988; McKeown-Eyssen et al. 1983) as well as animals (Gimenez-Llort et al. 2001; Rossi et al. 1997) reported greater developmental effects in males than in females, consistent with higher overall mercury retention and lower rates of mercury excretion by males. Although numerous factors that might differentially affect mercury disposition have been reported (Vahter et al. 2007), the biological mechanisms underlying sex-related differences in mercury excretion rates or susceptibility to mercury toxicity remain to be identified. Inasmuch as there are no known sex differences in humans with regard to the urine formation rate by the kidneys, the present findings imply a greater degree of mercury retention by males, possibly consistent with higher tissue levels observed in some studies.

Questions arise from the present observations as to the interpretation of urinary mercury concentrations in the assessment of safe mercury exposure levels. If girls are, in fact, more proficient in the excretion of mercury than boys, then it may follow that a specific urinary mercury concentration measured in girls represents a lesser risk of mercury toxicity than the same urinary concentration in boys. This issue speaks directly to the question of differential sensitivity (Brent and Weitzman 2004; Makre et al. 1986) and the establishment of precautionary measures directed at protecting the most susceptible from risks of mercury toxicity or mercury-associated disorders in children (U.S. Environmental Protection Agency 2000). Toxicokinetic studies that identify underlying sex-related differences in mercury handling and the use of metabolic biomarkers that reflect mercury body and tissue burden (Bowers et al. 1992; Pingree et al. 2001a; Woods 1995; Woods et al. 1993) may be useful in these endeavors.

In conclusion, in the present study we describe a strong, positive correlation between mercury exposure from dental amalgam fillings and urinary mercury excretion over a 7-year longitudinal course of amalgam treatment in children. However, significant differences in urinary mercury concentrations between boys and girls with comparable levels of amalgam treatment and times since placement suggest sex-related differences in mercury handling and, possibly, susceptibility to mercury toxicity. These findings are relevant within the context of children’s health risk assessment and suggest directions for future research to determine whether differential sensitivities to mercury between boys and girls do exist.

## Figures and Tables

**Table 1:** Demographics and baseline urinary mercury concentrations of the study participants by assigned treatment group.

Variable	Amalgam group (*n* = 253)	Composite group (*n* = 254)
Sex		
Female	116 (46)	112 (44)
Male	137 (54)	142 (56)
Race		
White	178 (70)	181 (71)
Black	75 (30)	68 (27)
Asian	0 (0)	5 (2)
Baseline age (years)	10.1 ± 1.0 (8.0–12.4)	10.0 ± 0.9 (8.2–12.0)
Baseline urinary mercury concentration (μg/L)	1.5 ± 1.2 (0.1–7.7)	1.4 ± 1.1 (0.0–8.6)

Values are no. (%) or mean ± SD (range).

**Table 2:** Categorization of the amount of amalgam treatment at baseline (within the first year after the initial visit) and during the follow-up period separately for male and female participants.

Baseline amalgam treatment (no. of surfaces)	Follow-up amalgam treatment (no. of surfaces)	Male [no. (%)]	Female [no. (%)]
0–4	0	13 (9.5)	11 (9.5)
	1–9	10 (7.2)	3 (2.6)
	> 9	4 (2.9)	4 (3.4)
5–9	0	11 (8.0)	14 (12.1)
	1–9	23 (16.8)	17 (14.7)
	> 9	13 (9.5)	10 (8.6)
> 9	0	18 (13.1)	13 (11.2)
	1–9	30 (21.9)	28 (24.1)
	> 9	15 (10.9)	16 (13.8)
Total	—	137 (100)	116 (100)

The amount of treatment during both time periods was similar for males and females.
